# The Structure and Mechanical Properties of the UHMWPE Films Modified by the Mixture of Graphene Nanoplates with Polyaniline

**DOI:** 10.3390/polym11010023

**Published:** 2018-12-24

**Authors:** Tarek Dayyoub, Aleksey V. Maksimkin, Sergey Kaloshkin, Evgeniy Kolesnikov, Dilus Chukov, Tat’yana P. Dyachkova, Irina Gutnik

**Affiliations:** 1 National University of Science and Technology “MISIS”, Moscow 119049, Russia; aleksey_maksimkin@mail.ru (A.V.M.); kaloshkin@misis.ru (S.K.), kea.misis@gmail.com (E.K.); dil_chukov@mail.ru (D.C.); 2 Tambov State Technical University, Tambov 392000, Russia; mashtatpetr@mail.ru (T.P.D.); anosowa_i_w@mail.ru (I.G.)

**Keywords:** UHMWPE, graphene nanoplates, polyaniline, films, mechanical properties

## Abstract

Highly oriented UHMWPE films were reinforced with functionalized graphene nanoplates (GNP). GNP was functionalized by deposition of polyaniline (PANI) on the GNP surface. The structure of GNP/PANI was studied by Raman spectroscopy, and the structure of xerogels and films based on UHMWPE was studied by DSC and SEM. PANI promotes the reduction of the GNP aggregation in the UHMWPE matrix and increases the degree of crystallinity due to heterogeneous crystallization. The new lamellar crystal structure has a high drawability. The highest value of the tensile strength 1330 MPa (an increase of 45%) was obtained with a filler content of 2 wt % GNP/PANI, and the highest value of Young’s modulus 41 GPa (an increase of 32%) was obtained with a filler content of 1 wt % GNP/PANI. The effect of GNP with PANI fillers on the dynamic mechanical properties of the UHMWPE films was discussed.

## 1. Introduction

Recently, polymer science has started to focus on providing the possibility for obtaining oriented flexible-chain polymer forms (fibers or films) with an enhanced mechanical performance. Usually, it is associated with the features of crystallization of flexible chain polymers [1], which mainly occurs by forming crystals on folded chains. The number of folded chains in amorphous regions of the polymer is too small (less than 20% of the total number of folded chains in the cross section of the crystal), which weaken the mechanical performance of the polymer. Therefore, increasing the proportion of load chains by the orientational drawing method of the polymer is considered as an effective way to enhance the mechanical performance of flexible-chain polymers [1,2], which will lead to the restructuring of folded crystals and forming crystals on the extended chains. In this way, the structural organization of the amorphous phase will be changed, based on the following features: the increasing degree of orientation, the straightening and the uniformity of tie molecules. In other words, optimizing the conditions of preparing the initial samples and of drawing plays an important role [3].

Ultra high molecular weight polyethylene (UHMWPE) is a polymer with a very simple chemical composition—only hydrogen and carbon, but the hierarchy of its structure at the molecular level makes it more complex [4]. UHMWPE is classified as a linear homopolymer [5]. UHMWPE has a very long molecular chain with two types of regions—crystalline (also called crystalline lamellae) and amorphous. The molecular forms in crystalline are ordered and sheetlike regions, whereas they are disordered in amorphous. UHMWPE has good mechanical, physical and tribological properties, such as: extreme hardness and durability, good chemical resistance, abrasion resistance, impact resistance, being easy to fabricate, a very low coefficient of friction and being a non-polarity polymer [6,7,8,9,10,11,12]. Commercially, the UHMWPE films are considered one of the most promising films that is comparable to Kevlar. Additionally, UHMWPE has a very low density (0.93–0.95 g/cm^3^), which makes it the best material in the films form in terms of the strength to weight ratio [13]. UHMWPE composites reinforced with different fillers materials are being developed for many applications, such as aerospace, industrial, and biomedical applications. One of the aims of UHMWPE composites is to enhance the mechanical performances of the virgin UHMWPE [14]. Nano-reinforcement of polymeric materials could lead to improving their mechanical properties, depending on the nanofillers distribution. If the interactions between the filler and the matrix of polymer were good, successful nanocomposites could be produced.

Graphene is a monolayer of sp^2^ -hybridized carbon atoms arranged in a two-dimensional lattice, and much attention is paid to it due to its excellent thermal, mechanical and electrical properties [15]. It is considered an extremely stiff material, because it has a significant ultimate tensile strength of 130 GPa, a Young’s modulus of 1 TPa and zero effective mass [15]. All of these properties make graphene a more preferable material over carbon fillers in polymer composites. Due to graphene’s flat structure, its distribution in polymeric matrix at different directions is better than other carbon fillers. A lot of studies that used graphene as filler in polymeric nanocomposites have shown an improvement in mechanical properties [16,17]. Graphene platelets have a rich surface in reactive functional groups that play an important role in the bonds between graphene platelets and polymer matrices [18,19]. Graphene nanoplates show aggregation caused by van der Waals interactions between individual sheets, and that leads to a relatively low surface area. Therefore, polyaniline could be used together with graphene as a material with pseudocapacitance to increase the specific surface area of GNP [20,21,22]. Polyaniline was chosen for GNP encapsulation for cost reduction, dispersion and compatibility in polyethylene improvement [23,24]. Addiego et al. reported that PANI acts as an acceptor of free radicals in the UHMWPE fabrication and does not impair the mechanical properties of the material [25].

Ni et al. prepared the UHMWPE/GO (graphene oxide) composites by using liquid-phase ultrasonication mixing followed by hot-pressing. In comparison with the virgin UHMWPE, they found that the crystallinity was improved with the increase of the GO content. Also, they reported that the tensile strength had an optimal value at the GO content of 0.5 wt % with an increase of 12%, and the tensile strength at the GO content of 1 wt % had increased by 1% [26]. Bhattacharyya el al. prepared the composites of UHMWPE with reduced graphene oxide in two methods, which are the pre-reduction method and the in situ reduction method. They reduced the graphene oxide by phenylhydrazine. They found that the strength value in the pre-reduction method (22 MPa) is much better than the in situ reduction method (17 MPa) due to the reinforcement caused by graphene as well as a higher crystalline content (an increase of 76% in comparison with virgin UHMWPE (13MPa)) [27]. Yan et al. prepared the UHMWPE/GO composites by liquid-phase ultrasonication dispersion followed by hot-pressing. They reported that these composites exhibited a good biocompatibility; and the composite of 0.5 wt % graphene oxide had the best ultimate tensile strength (31 MPa) in comparison with the virgin UHMWPE (30 MPa). Nevertheless, they found that the composites of 0.1, 0.3, 1 wt % graphene oxide had an ultimate tensile strength that was less than the ultimate tensile strength of the virgin UHMWPE (23, 24, 27 MPa, respectively) [28]. Achaby et al. compared the mechanical properties of HDPE/GNs and HDPE/MWCNTs nanocomposites, which were prepared by using the method of melt blending. They found that the tensile strength of the HDPE/graphene nanocomposite with a graphene content of 3 wt % (47 MPa) was higher than the virgin HDPE (26 MPa) by 77%. Also, they reported that the increase of 58% was found in the case of the HDPE/MWCNTs nanocomposites (41 MPa) at the same filler content [29].

In this study, bulk oriented UHMWPE films were reinforced with graphene nanoplates (GNP) and with functionalized graphene nanoplates by polyaniline (PANI). Xerogels of UHMWPE/GNP and UHMWPE/GNP/PANI have been drawn and oriented to an average drawing ratio (DR) of 60. The prepared bulk oriented films UHMWPE/GNP and UHMWPE/GNP/PANI were studied. They showed an improvement in the tensile properties and a good adhesion between GNP/PANI and UHMWPE; these properties are to be considered in designing the composite materials. The UHMWPE films have a light weight, a slippery surface and a high strength, which make them an ideal choice for the industrial applications like lining and coatings for equipment that require sliding surfaces, or producing some sports parts of a high quality. Also, UHMWPE films can be applied in many biomedical applications.

## 2. Materials and Methods

### 2.1. Materials

UHMWPE with molecular weight of 1 × 10^6^ g/mol was purchased from Kazanorgsynthesis Ltd (Kazan, Russia). Graphene nanoplates (GNP) was obtained by oxidative intercalation of expanded graphite with subsequent ultrasonic treatment was purchased from Nanotechcenter Ltd (Tambov, Russia).

### 2.2. Graphene Nanoplates Functionalization

**Figure 1:**
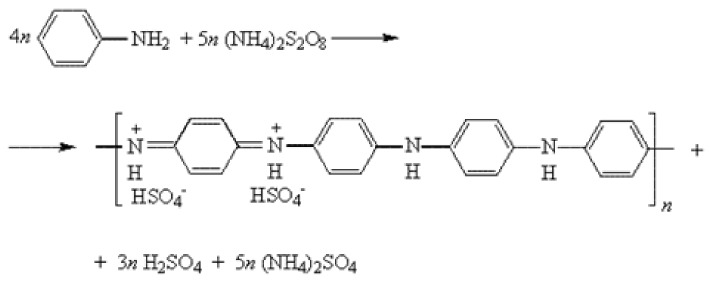
The scheme of oxidative polymerization of aniline.

Filler is functionalized by deposition of polyaniline (PANI) on the graphene nanoplates surface. Previously, 2 g GNP was dispersed in distilled water (500 mL) by ultrasonic treatment. Concentrated hydrochloric acid was added to the suspension in an amount, which was necessary for the initial level of pH = 1. Then aniline (1.28 g, 2.76 × 10^–2^ mol/L) and ammonium persulfate (2.62 g, 3.45 × 10^–2^ mol/L) were added. The reaction mass was stirred for 5 h at room temperature. The formation of PANI occurred as a result of the oxidative polymerization of aniline according to the scheme, which is shown in Figure 1. At the end of the reaction, the product was separated by filtration, and successively washed by distilled water and isopropyl alcohol until the color of the filtrate had disappeared and then dried at 80 °C. The PANI content in the obtained material was determined by the gained weight to the mass of the initial GNP. The mass of the resulting material sample was 3.3 g, so the PANI content was about 40 wt %. 3 samples by this procedure were produced with a stable PANI content.

According to the reference [30], there are different types of oxygen–containing groups (–C–O–, –C=O, –COOH) on the surface of GNP, which were obtained by chemical exfoliation of graphite. These groups can interact with PANI macromolecules by electrostatic attraction and form hydrogen bonds [31,32]. Depending on the method of molecular dynamics, by the simulation results of the PANI interactions with oxidized carbon nanostructures (GNP and carbon nanotubes) [33], a carboxyl group has a directing influence on the position of the PANI macromolecules in the modifying layer, and they bond together through π-π-electron interaction and the forces of van der Waals.

### 2.3. Fabrication of Films

The mixing of GNP and GNP/PANI with UHMWPE powder was done by using a high-energy planetary ball mill APF-3 in steel drums that have a volume of 900 mL. Steel balls (Russian grade SHKH15) with a diameter of 7 to 9.5 mm were used as grinding media. The temperature of the samples was controlled in the milling process by using current water to cooling the steel drums, so that the temperature inside the drums did not exceed 80 °C. The average rotation speed of the carrier was 450 rpm. The total mixing time was 60 min. The UHMWPE/GNP/PANI composite powders with various filler contents (0.1, 0.5, 1.0, 1.5, 2.0 wt %) were prepared. The UHMWPE/GNP composite powders were prepared with the same filler content as control samples.

UHMWPE has a very high melt viscosity, so that it is difficult to prepare UHMWPE xerogels with a good ability to multi-stage drawing by using the melt under extrusion technology. Therefore, the gel-spinning technology was used [34] in order to obtain high-strength materials based on UHMWPE. Previously, Maksimkin et al. developed a new approach for fabricating bulk- oriented films based on UHMWPE by using a small amount of solvent as a plasticizer [35].

P-xylene for plasticizing UHMWPE with a ratio of 2.5 mL of solvent per 1 g of UHMWPE was used. After storage for 15 min at 143 ± 3 °C, the UHMWPE/p-xylene gel was fabricated at the same temperature, and then extruded by ram extruder UE-MSL (Extrusion Machinery Sales Ltd, Liversedge, UK). The die size was 10 mm × 2 mm and the extrusion rate was equal to 500 mm/min, and the extruded gel was dried for 48 h at room temperature. After that, the first step of orientation of extruded gel was accomplished by using rolling machine BL-6175-A. Dried gel or xerogel (solvent-free UHMWPE gel) was rolled at 100 °C to reach 2–2.5 draw ratio value. Therefore, each UHMWPE/GNP/PANI composites has a different draw ratio value in the range of 2–2.5. In the next step, a special developed and fabricated laboratory device (equipment) consisting of stepping motors and sheaves set (system) was used to pass the dried UHMWPE film through bath with silicone oil and to draw the films. The oil temperature was stable within ±0.1 °C precision. Multi-stage of hot orientation for the UHMWPE films was carried out stepwise at various temperatures: 110 °C to DR = 10–12; 120 °C to DR = 19–21; 130 °C to DR = 28–30 and 140 °C to DR = 57–64. In each stage of the orientation, each composite has a different range of draw ratio values at each temperature.

### 2.4. Testing Procedures

Tensile strength of oriented UHMWPE films was measured according to ASTM D882-10 using Zwick/Roell Z020 universal testing machine at 10 mm/min loading rate. At least 5 measurements were applied for each sample. The surface of the films was cleaned by acetone before testing to remove the silicone oil, which could be remained from the drawing step. The UHMWPE films have a very low friction coefficient, which creates difficulties in their mechanical tests. When the films are fixed in the grips of the test machine and the test starts, the films may slip out of the grips. The increase of the clamping pressure of the grips leads to premature failure in the grip area. Therefore, prior to mechanical tests, both ends of the films were glued to thin cardboard with a size of 60 × 50 mm^2^. Tests of the films were carried out using clamping jaws with thin notches, to ensure their reliable fastening for films without creating a local stresses concentration in the captures area. Slips of the samples during the test were not observed.

The structure of UHMWPE xerogels and films was studied by using scanning electron microscope JEOL JSM-6610LV at accelerating voltage of 20 kV. To avoid a charge accumulation, the polymer surface was coated with a Pt layer of 10–20 nm in thickness (magnetron deposition equipment JFC-1600 was used, JEOL Ltd, Tokyo, Japan). At least 3 samples for each composite were studied. To obtain a fracture surface for the UHMWPE xerogels, the sample was stored in a liquid nitrogen (T = 77 K) for 20 min and then was broken into two halves. The lateral surface of the oriented structure of the UHMWPE films was obtained by the tearing mechanism in the direction of the orientation.

Crystallinity and melting temperature of the films were determined by differential scanning calorimetry (DSC) (NETZSCH DSC 204 F1, NETZSCH Group, Selb, Germany) at a heating rate of 10 °C min^−1^ from 35 to 180 °C in argon atmosphere according to the ASTM D 3417-83. At least 3 samples for each composite were analyzed. The relative degree of crystallinity was calculated as the ratio of the experimental sample melting enthalpy to the completely crystallized polyethylene melting enthalpy, which is equal to 293 J/g [36]. The key parameters for DSC curves processing were T_m_^onset^–the onset of the melting peak, T_m_—the melting temperature peak and T_m_^end^—the end of the melting peak.

Dynamic mechanical analysis was carried out by DMA Q800 (TA Instruments, New Castle, DE, USA). The samples were analyzed in a single cantilever bending mode. The specimens of dimensions 60 × 3.24 × 0.16 mm^3^ were heated from 25 to 160 °C at the heating rate of 3 °C min^−1^. The samples with free ends had bended in one-point at a prespecified 10 μm amplitude and 1 Hz oscillation frequency.

Raman spectroscopy of GNP/PANI powder was carried out by Thermo Scientific DXR using the laser wavelength of 633 nm.

## 3. Results and Discussion

**Figure 2:**
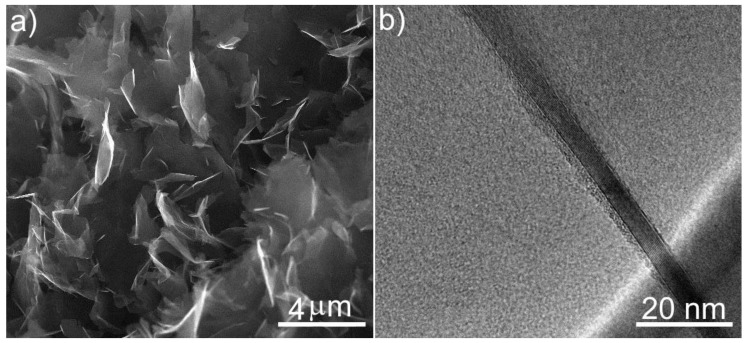
(**a**) SEM and (**b**) TEM micrographs of GNP microstructure.

Figure 2a,b shows the SEM and TEM images of the initial GNP. The size of the nanoplate in the sheet is 2–10 μm and the thickness of nanoplates is 6–8 nm. GNP are multilayers and consist of 15–25 of graphene layers.

**Figure 3:**
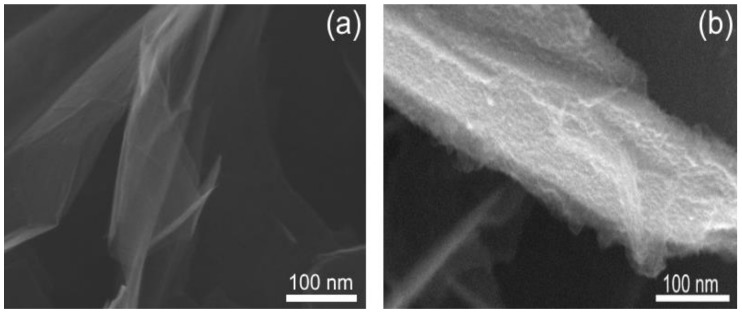
SEM micrograph of the (**a**) unmodified GNP and (**b**) modified GNP by PANI.

**Figure 4:**
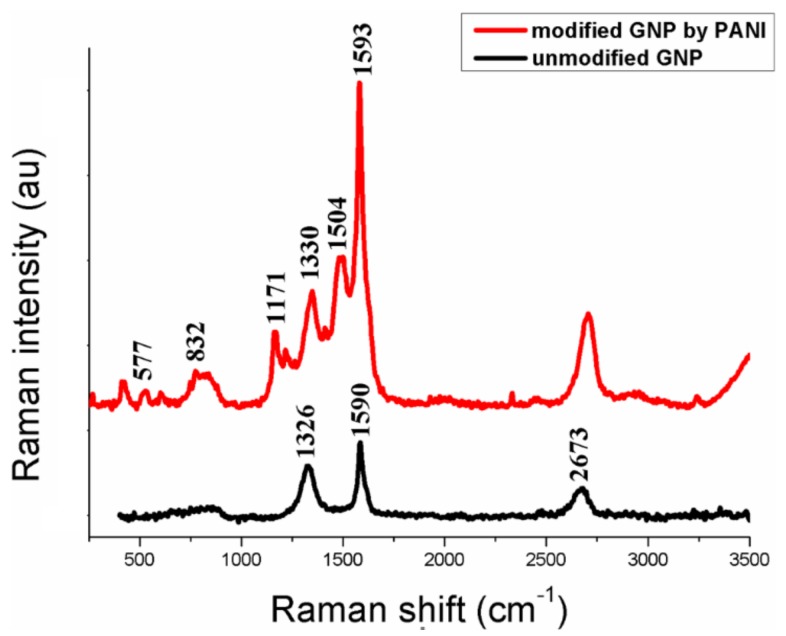
Raman spectra of (**a**) unmodified GNP and (**b**) modified GNP by PANI.

Figure 3 demonstrates the SEM of GNP after functionalization by PANI. It can be seen that the structure of PANI layer on the GNP surface is a knobby structure. The presence of PANI on the GNP surface was confirmed by Raman spectroscopy (Figure 4). Raman spectra for unmodified GNP showed three typical broad peaks at ~1326, 1590 and 2673 cm^−1^, ascribed to D, G and 2D modes, respectively. The peaks at 1593, 1504, 1330, 1171, 832, 577 cm^−1^ are an indication of the presence of PANI as the protonated form of the emeraldine salt [37].

**Figure 5:**
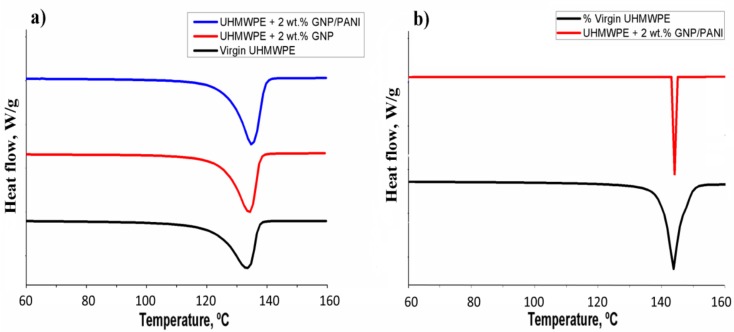
DSC test results for: (**a**) UHMWPE xerogels, (**b**) UHMWPE films.

The results of DSC test for the xerogels are presented in Table 1. The average values of the three samples, characterized by DSC for each composition were used. The standard deviation for the temperature values was ± 0.1 °C. Figure 5a shows the DSC thermograms obtained for some of the UHMWPE xerogels (virgin UHMWPE, UHMWPE with 2 wt % GNP and UHMWPE with 2 wt % GNP/PANI). As can be seen in Table 1, the crystallinity of the virgin UHMWPE xerogel (70 ± 2%) has been increased up to 87 ± 2 % after the GNP/PANI addition. On the other hand, the effect of the GNP addition without PANI on the crystallinity of xerogels was not observed.

The PANI plays an important role in the crystallization processes of UHMWPE macromolecules for several reasons. First, it could be connected to the presence of oxygen-containing groups on the surface of the GNP covered by PANI macromolecules (chains), so that the functionalized GNP by PANI is better combined with the non-polar matrix [23,38]. In addition, the granular morphology of PANI on the modified surface of the GNP could be important factor in the crystallization processes of UHMWPE macromolecules. As a result, the formation of heterogeneous nucleating sites for crystallization of macromolecules may occur, leading to an increase in the crystallinity of the UHMWPE.

The melting temperature for polymers depends mainly on the crystalline phase size. According to the Thomson-Gibbs equation [39], small and defected crystallites have a lower melting temperature compared to larger size and non-defected crystallites. The crystalline phase size and the degree of xerogels crystallinity have therefore increased in the UHMWPE xerogel as a result of heterogeneous crystallization. In the reference [28], it was reported that when the graphene oxide was added into the polymer matrix, the graphene oxide surface could act as nucleation sites for the crystallization. Therefore, the nucleation and growth of individual crystallites were very fast, so that a very large number of crystallites were formed together.

**Figure 6:**
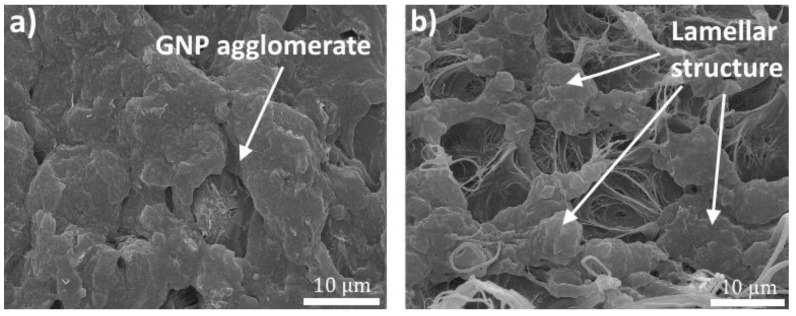
SEM micrographs showing typical supramolecular structure of the UHMWPE xerogels with (**a**) GNP and (**b**) GNP/PANI.

**Figure 7:**
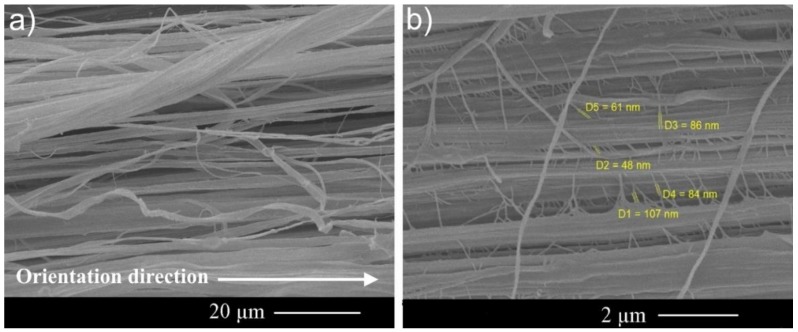
Typical fibrillar structure of the UHMWPE films with different magnifications. (**a**) A well-defined fibrillar structure in the UHMWPE films, (**b**) A large number of perpendicular nanofibrils to the oriented fibrils of UHMWPE with diameters in the range of 50–100 nm.

Figure 6 shows a typical supramolecular structure of the UHMWPE xerogels with GNP and GNP/PANI. The tested surface was created by a quasi-brittle fracture. The supramolecular structure of the UHMWPE/GNP/PANI xerogels is a lamellar crystalline structure (Figure 6b). These structures have a high drawability and during the quasi-brittle fracture some of the lamellar crystals were transformed into fibrils (see Figure 7). GNP agglomerate in UHMWPE was observed (Figure 6a). It should be noted that in our previous work a similar method of nanofiller distribution in UHMWPE was used, but agglomerates at the nanofillers content of 1–2 wt % were observed [40]. This confirms that the functionalization by PANI reduces the aggregation of the GNP in UHMWPE.

Table 2 presents the results of the mechanical tensile tests of the oriented UHMWPE films with different filler contents. The average values of the five samples for each composition were used. It was found that the GNP addition without PANI provides a decrease in the tensile strength and Young’s modulus for the UHMWPE/GNP films. In the reference [28], they found that the tensile strength of UHMWPE/GO composites was lower than the tensile strength of the virgin UHMWPE. They reported that the tensile strength had an optimal value when the GO content was 0.5 wt % and too high or too low GO content would reduce the performance of materials. But the GNP/PANI addition leads to a significant increase in mechanical properties. At low GNP/PANI contents (0.1 and 0.5 wt %), the tensile strength has increased. It peaked at a content of 2 wt % (an increase of 45% compared to the virgin UHMWPE films). The maximum Young’s modulus (41 GPa) was detected by filling 1 wt % of GNP/PANI (an increase of 32%). The elongation at break for all the UHMWPE films was in the range of 4.8–7%.

The results of DSC test for the films are presented in Table 3. The average values of the three samples, characterized by DSC for each composition were used. The standard deviation for the temperature values was ± 0.1 °C. Figure 5b shows the DSC thermograms obtained for some of the UHMWPE films (virgin UHMWPE and UHMWPE with 2 wt % GNP/PANI). As can be seen in Table 3, all the UHMWPE films have a high crystallinity up to 95–97% because of the orientation of the UHMWPE amorphous phase and the deployment of lamellae to the drawing direction and their recrystallization. These processes are proceeding simultaneously with the reduction of crystalline phase defects. Compared to the xerogels, the melting temperature of the UHMWPE films had increased, which explains the increase of the size of crystalline phase and the reduction of crystalline phase defects. It should be noted that Δ*T*_m_^film^ (*T*_m_^end^ − *T*_m_^onset^)_film_ < Δ*T*_m_^xerogel^ (*T*_m_^end^ − *T*_m_^onset^)_xerogel_, and *T*_m_^film^ > *T*_m_^xerogel^. In Table 1 and Table 3, for the virgin UHMWPE, Δ*T*_m_^film^ < Δ*T*_m_^xerogel^ (11.9 °C < 14.3 °C) and *T*_m_^film^ > *T*_m_^xerogel^ (143.5 °C > 133.3 °C); and also for the UHMWPE/GNP/PANI of 2 wt % GNP/PANI, Δ*T*_m_^film^ < Δ*T*_m_^xerogel^ (8.2 °C < 12.2 °C) and *T*_m_^film^ > *T*_m_^xerogel^ (144.1 °C > 135.0 °C). It could be explained that the increase of T_m_^films^ is related to the new crystallization zone formed by the extended molecular chains of the films, which requires a higher melting temperature. The process of drawing had transformed the folded-chain lamella structure of the UHMWPE xerogels into high-oriented extended-chain crystals (fibrils). Fibrils have shown narrower accumulation and a higher melting point related to their massive internal stress than the lamella structure. According to the aforementioned characteristics, since the range of the melting temperature related to the polymers films is narrower than the one related to the polymers xerogels, the crystals in films are formed and grown at the same time.

Crystallization of the UHMWPE macromolecules on the GNP/PANI leads to the formation of lamellar structure with a high drawability. As a result, the process of multistage hot orientation for UHMWPE films is considered to be the simplest and films with higher DR (Table 2 third column) can be obtained. Although the degree of crystallinity of all UHMWPE films reaches up to high values (95–97%), the simplification of multistage hot orientation processes helps to reduce the number of breaks in polyethylene macromolecules during the drawing, and to have higher values of the tensile strength and Young’s modulus. On the other hand, the crystallization of the UHMWPE macromolecules on the GNP/PANI promotes the deep integration of the filler into the polymer, which increases the load transfer between the fillers and the polymer matrix.

Figure 7a,b shows SEM images of typical lateral surface of the UHMWPE films. Figure 7a indicates a well-defined fibrillar structure in the UHMWPE films. There are a large number of perpendicular nanofibrils to the oriented fibrils of UHMWPE that have diameters in the range of 50–100 nm (Figure 7b). As can be seen, they play an important role in connecting oriented fibrils, so that there will be no possibility for breaking the films into separate fibrillary components.

**Figure 8:**
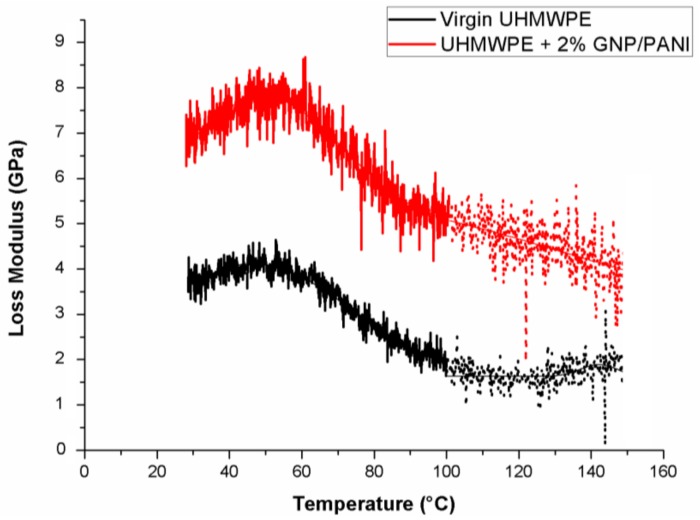
DMA dynamic loss modulus of the UHMWPE/GNP/PANI films.

The α-relaxation of the UHMWPE is the result of deformation or movements within the interfacial regions (folds or tie molecules), which were activated because of chain mobility in the crystalline region [41]. The α-relaxation temperature of the UHMWPE is very wide, ranging between 30 and 120 °C [42], depending on the crystallite thickness. The thicker crystals (lamellae) have led to an increase to the α-relaxation temperature values [43]. The effect of the various filler contents promoting an influence on the α-relaxation of UHMWPE with GNP/PANI films is shown in Table 4. The α-relaxation temperature values are evaluated at the maximum peaks of the loss modulus curves by measuring the FWHM (Full Width of Half Maximum). Figure 8 shows the effect of the 2 wt % GNP/PANI in the α-relaxation. The values of T_α_ are very close to each other, so the lowest temperature of α-relaxation was observed for the film of 2 wt % GNP/PANI. The α-relaxation of polyethylene had decreased with a lower crystallite size [42].

According to ISO 6721-1, the loss modulus is the measure of the energy dissipated during one loading cycle, representing the viscous response of a material. This is related to the stress and the elongation in the materials. As can be seen in Figure 8, the loss modulus of the UHMWPE/GNP/PANI films of 2 wt % GNP/PANI was higher than the loss modulus of the virgin UHMWPE. This could be explained by the prevalent viscous behavior of the UHMWPE/GNP/PANI films. Therefore, modifying the UHMWPE by the GNP/PANI could increase the mechanical restraint of the materials, leading to a decrease in the flexibility of the UHMWPE/GNP/PANI films [43].

**Figure 9:**
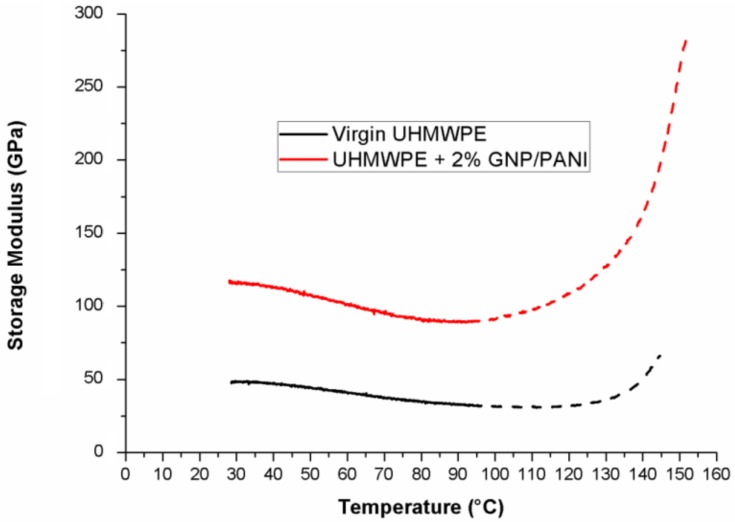
DMA dynamic storage modulus of the UHMWPE/GNP/PANI films.

The value of storage modulus indicates the material’s ability to store the energy of external forces without permanent strain deformation. Therefore, a higher storage modulus is associated with a higher elastic property of material [44], and therefore, to its stiffness. As shown in Figure 9 and Table 4, the highest storage modulus at room temperature was observed for the UHMWPE/GNP film of 2.0 wt % GNP/PANI, showing a significant increase in stiffness. Good uniform distribution [31,32,33,34,35,36,37,38,39,40,41,42,43,44,45,46] and a strong interfacial interaction between a polymer matrix and a filler lead to an increase in the storage modulus of polymers. Also, in Figure 9, it can be seen that above the temperature of 100 °C, an increase in the storage modulus can be observed in all films (Figure 9, discontinuous lines). The highest value of storage modulus for all samples was observed at a temperature of about 153 °C. This effect is associated with the shape memory effect (SME) in the UHMWPE [47]. When the temperature of the sample increases (the energy is given to samples), the SME in the UHMWPE matrix is activated. As a result, the sample begins to return to its original form (form before orientation) and an increase in the cross section of the sample occurs, resulting in an apparent increase in the storage modulus.

**Figure 10:**
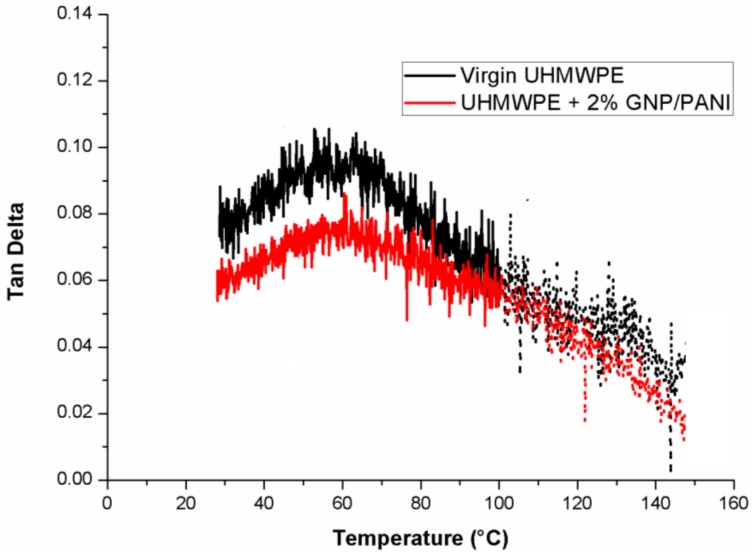
DMA tanδ for the UHMWPE/GNP/PANI films.

One of the important properties of polymer composites is the damping response, i.e., how efficiently the composite loses energy by molecular rearrangements, which in this case is indicated by the values of tan δ. Tan δ represents the ratio of the viscous to elastic response of a viscoelastic material or in another words the energy dissipation potential of the material. As it can be seen in Figure 10, the tan δ values for the virgin UHMWPE films and the UHMWPE/GNP/PANI of 2 wt % GNP/PANI films are ranging from 0.06 to 0.075 at room temperature. The lowest value of tan δ at room temperature for the UHMWPE/GNP/PANI films was obtained for 2.0 wt % GNP/PANI. This means that the composites behave in a more elastic way. They have the potential to store the load rather than dissipating it by applying a load. The tan δ values, proceeding their decrease to 0 by being heated to a degree above 100 °C, are associated with the shape memory effect (SME) in the UHMWPE [47]. Therefore, the sample begins to return to its original form (form before orientation) and an increase in the cross section of the sample occurs, resulting in an apparent decrease in the tan δ values.

## 4. Conclusions

In this paper, the GNP/PANI mixture was used to improve the mechanical properties of the highly oriented UHMWPE films. The GNP was functionalized by PANI using the oxidative polymerization of aniline on the GNP surface to improve filler/matrix interaction. It was found that the structure of PANI layer on the GNP surface is a knobby structure and appears as the protonated form of the emeraldine salt. The GNP/PANI behaves as a nucleating site for crystallization of the UHMWPE macromolecules. The PANI plays an important role on the crystallization processes of the UHMWPE macromolecules. Although GNP has a large specific surface area, the GNP without PANI practically has no effect on the degree of crystallinity of the UHMWPE xerogels. The heterogeneous crystallization of the UHMWPE macromolecules on the surface of GNP/PANI leads to the increase of the crystallinity degree of the xerogels, and to the formation of a lamellar crystalline structure with high drawability. High drawability of the UHMWPE xerogels facilitates the multistage hot orientation of the films, and reduces the number of breaks in polyethylene macromolecules during the drawing. As a result, the UHMWPE/GNP/PANI films with a higher draw ratio and higher mechanical properties in comparison with the virgin UHMWPE films were obtained. Additionally, the crystallization of the UHMWPE macromolecules on the GNP/PANI promotes a deep integration of the filler into the polymer, which increases the load transfer between the filler and the polymer matrix. Due to these factors, the tensile strength of the oriented UHMWPE films has increased by 45% at the GNP/PANI content of 2 wt % and Young’s modulus has increased by 32% at the GNP/PANI content of 1 wt %

In addition, the values of T_α_ for the UHMWPE/GNP/PANI films have decreased in comparison with the virgin UHMWPE film, which means a lower crystallite size and more stable crystals for the UHMWPE/GNP/PANI films. It was shown that the values of storage modulus for the UHMWPE/GNP/PANI films were increased in comparison with the virgin UHMWPE film and the highest value for the UHMWPE/GNP/PANI film was obtained for 2 wt % GNP/PANI. Also, as shown, the values of loss modulus for the UHMWPE/GNP/PANI films had increased in comparison with the virgin UHMWPE films, which means that there had been a decrease in the flexibility of the UHMWPE/GNP/PANI films. It was shown that the shape memory effect (SME) in the UHMWPE film can be activated at a temperature above 100 °C, which results in the increase in cross section of the samples and in the apparent increase in storage modulus. It was found that the UHMWPE/GNP/PANI films are more elastic in comparison with the virgin UHMWPE film and the lowest tan δ value at room temperature for the UHMWPE/GNP/PANI film was obtained for 2 wt % GNP/PANI.

## Figures and Tables

**Table 1:** DSC test results for the UHMWPE xerogels (± the standard deviation).

Material	Filler content, %	T_m_^onset^, °C	T_m_, °C	T_m_^end^, °C	Crystallinity, %
**UHMWPE**	-	123.0	133.3	137.3	70 ± 2
**UHMWPE/GNP**	0.1	125.2	133.2	136.0	69 ± 2
	0.5	129.7	138.7	142.3	72 ± 2
	1.0	128.2	138.3	142.2	68 ± 2
	1.5	125.2	133.7	137.2	70 ± 2
	2.0	125.7	134.2	137.7	75 ± 2
**UHMWPE/GNP/PANI**	0.1	125.5	134.5	138.1	79 ± 2
	0.5	125.3	136.1	140.1	86 ± 2
	1.0	123.8	134.2	137.7	76 ± 2
	1.5	126.3	136.0	140.8	84 ± 2
	2.0	127.2	135.0	139.4	87 ± 2

**Table 2:** Mechanical tensile properties of the UHMWPE films with different filler content (± the standard deviation).

Material	Filler content, %	DR	Young’s modulus, GPa	Tensile strength, MPa	Elongation, %
**UHMWPE**	-	35	31 ± 5	920 ± 13	7.0 ± 1
**UHMWPE/GNP**	0.1	34	26 ± 2	803 ± 45	4.8 ± 0.4
	0.5	33	28 ± 3	833 ± 42	6.7 ± 0.5
	1.0	35	28 ± 2	843 ± 40	9.0 ± 2.0
**UHMWPE/GNP/PANI**	0.1	62	38 ± 5	1190 ± 79	6.6 ± 1.1
	0.5	61	35 ± 4	1220 ± 130	5.7 ± 0.5
	1.0	64	41 ± 4	1110 ± 120	4.9 ± 0.8
	1.5	64	32 ± 3	1050 ± 35	5.8 ± 0.7
	2.0	57	32 ± 5	1330 ± 77	6.8 ± 0.7

**Table 3:** DSC test results for the UHMWPE films (± the standard deviation).

Material	Filler content, %	T_m_^onset^, °C	T_m_, °C	T_m_^end^, °C	Crystallinity, %
**UHMWPE**	-	138.4	143.5	150.3	94 ± 2
**UHMWPE/GNP/PANI**	0.1	138.7	142.3	144.5	95 ± 2
	0.5	142.6	144.9	146.6	95 ± 2
	1.0	138.7	144.0	146.4	97 ± 2
	1.5	142.1	145.1	147.6	97 ± 2
	2.0	138.1	144.1	146.3	97 ± 2

**Table 4:** Dynamical mechanical properties of the UHMWPE/GNP/PANI films at room temperature and the α-relaxation temperature evaluated at the maximum peaks of the loss modulus curves.

Material	Filler Content, %	Storage Modulus, GPa	tan δ	α-Relaxation, °C
**UHMWPE**	-	54.67	0.075	47.8
**UHMWPE/GNP/PANI**	0.1	60.64	0.071	47.1
	0.5	70.91	0.070	46.8
	1.0	81.02	0.069	46.7
	1.5	92.55	0.065	46.2
	2.0	120.93	0.060	46.1
